# Prevalence of target anaerobes associated with chronic periodontitis

**DOI:** 10.1099/acmi.0.000177

**Published:** 2020-11-16

**Authors:** Ameerah M. Alazemi, W. Jamal, A. Al Khabbaz, V. O. Rotimi

**Affiliations:** ^1^​ Microbiology Department, Faculty of Medicine, Kuwait University, Kuwait; ^2^​ Department of Surgical Sciences, Faculty of Dentistry, Kuwait University, Kuwait

**Keywords:** Anaerobes, etiology, Kuwait, periodontal infections

## Abstract

**Introduction:**

Periodontal diseases are a group of chronic infections that destroy tissues surrounding and supporting the teeth. Data on the anaerobes associated with periodontal infections in Kuwait is lacking.

**Aim:**

To investigate the target anaerobes associated with chronic periodontitis (CP) in patients admitted to Dental Clinics in Kuwait University Health Sciences Center, Kuwait.

**Methodology:**

Patients with CP (severe and moderate) were recruited into this study during a period of 15 months. Samples were collected directly from inside the gingival pockets and subjected to semi-quantitative PCR assays.

**Results:**

A total of 30 patients, stratified into moderate and severe CP and 31 healthy individuals, used as controls, were studied. Nine (30 %) of the 30 patients were in the 50–59-year age group. The detection rate of *Aggregatibacter actinomycetemcomitans* between the patients (9 : 30 %) versus the controls (5 : 16.1 %) was non-significant (*P >*0.05). *Fusobacterium* spp., were detected in all patients versus 29 (93.1 %) controls, (*P >*0.05). However, four target anaerobes were significantly associated with CP patients; *Porphyromonas gingivalis* was detected in ten (33.3 %) patients versus two (6.4 %) controls (*P* <0.0001); *Tannerella forsythia* 25 (83.3 %) versus 16 (51.6 %) controls (*P* <0.0001); *Parvimonas micra* 27 (90 %) versus 16 (51.6 %) controls (*P* <0.0001) and *Treponema denticola,* 18 (60 %) versus nine (29 %) controls (*P* <0.0001), respectively. *Prevotella* spp. were detected in 27 (90 %) patients and 30 (96.7 %) controls (*P*>0.5). There was no significant difference in the burden of *Prevotella* spp. between patients and controls determined by semi-quantitative PCR assays.

**Conclusion:**

Some (4/7) of the target anaerobes were significantly associated with CP in our study. *P. gingivalis* was the most strongly associated anaerobe with CP, although not the keystone bacteria, while *Prevotella* spp. was similar to the healthy controls.

## Introduction

Periodontal diseases are chronic infectious diseases in which the pathogenic micro-organisms in the subgingival biofilm initiate immune response in the host and pathogens possess many tissue-destructive virulence factors of their own leading to destruction of tooth supporting tissue and if untreated, can lead to tooth loss [1]. Chronic periodontitis (CP) is the most common type of periodontitis and is often a slowly progressive disease that becomes apparent in adulthood and continues for the rest of the life of the patient [1].

This disease is highly prevalent all over the world. It has multifactorial contributing factors such as local, systemic, environmental as well as genetic factors. An estimated count of the microbiota of the oral cavity is over 700 bacterial species [1]. However, certain anaerobic bacteria, *Porphyromonas gingivalis *, *Tannerella forsythia* and *Treponema denticola,* the so-called ‘red complex’, have been significantly associated with chronic periodontal diseases (CPDs) [[2]]. According to some published reports other species such as *Fusobacterium* spp.*, Prevotella* spp.*, Campylobacter rectus*, *Eubacterium nodatum, Parvimonas micra* and *Aggregatibacter actinomycetemcomitans* are also considered to be closely associated with periodontitis [2]. However, the mechanism of CPD and destruction of the surrounding bone is thought to be mediated by the host response to a unique bacterial consortium and not just due to a single pathogen [1].

Accurate detection of the potential periodontal pathogens in the periodontal pockets, subgingival plaque or crevicular fluid samples is often dependent on the method used. Numerous qualitative and quantitative methods have been used for the identification of putative periodontal pathogenic micro-organisms, including culture or culture-independent methods. Most of the oral pathogens are non-cultivable, and this is the rational for use of molecular detection methods such as PCR. Several molecular techniques such as PCR-based methods have been used to detect the potential periodontal pathogens.

There seems to be a wide variation in the prevalence and severity of the disease in different developed and developing countries and in different geographic regions [3, 4]. This variation may be related to the aetiology of the disease, demographic and cultural factors [3]. Hence, it is important to obtain information about the anaerobes that are associated with CPD in various locations. The objective of this study was to investigate the seven target anaerobes associated with chronic periodontitis in patients admitted to different dental units in Kuwait University Health Sciences Center and in periodontally healthy patients in Kuwait.

## Methods

### Study population

An active prospective surveillance of patients with chronic periodontitis was initiated in December 2015 and completed in April 2017 at the Dental Clinic (DC), Kuwait University Health Sciences Center (KUHSC), Kuwait. Thirty patients with periodontal diseases aged between 20–70 years were recruited. These patients were carefully examined and categorized into two groups; moderate CP (MCP) and severe CP (SCP) by a single well-trained and competent consultant (AAK) throughout the study period. Biodata including age, sex, nationality, history of antibiotic use in the last 6 months and co-morbidity were carefully documented.

Moderate disease was defined as 3–4 mm of attachment loss between the cemento-enamel junction to the bottom of periodontal pocket and severe disease as ≥5 mm of attachment loss (see Table 1) [5]. Thirty-one healthy individuals, without periodontal disease, who attended the clinic for regular checkup, were included as control. Inclusion criteria included the following: medically healthy adult patient with minimum age of 18 years, could go under routine dental examination without the need of antibiotic prophylaxis, no history of antimicrobial therapy or/and dental prophylaxis in the last 6 months and absence of orthodontic appliances.

**Table 1.** Guidelines for determining severity of periodontitis

	Slight (mild)	Moderate	Severe (advanced)
Probing depths	>3 and <5 mm	≥5 and <7 mm	≥7 mm
Bleeding on probing	Yes	Yes	Yes
Radiographic bone loss	Up to 15 % of root length or ≥2 mm and ≤3 mm	16–30 % or >3 mm and ≤5 mm	>30 % or >5 mm
Clinical attachment loss	1 to 2 mm	3 to 4 mm	≥5 mm

According to American Academy of Periodontology [5].

Exclusion criteria included the following: patients presenting with less than 20 teeth or diagnosed with aggressive periodontitis, history of periodontal treatment or antibiotic/oral antiseptic use in the previous 6 months, pregnancy or breast feeding, and any drug intake known to modify periodontal inflammation, such as anti-inflammatory drugs.

### Microbiological sampling

Duplicate subgingival plaque samples were taken from sites by carefully removing supra-gingival plaque with curettes and cotton pellets before inserting, size 60, 6–8 sterile endodontic paper points in periodontal pocket and leaving them *in situ* for 10 s. The points were immediately transferred to sterile, dry micro-centrifuge tubes. To avoid PCR inhibition by components present in blood, excessively blood-soaked points were discarded and new samples taken from a less haemorrhagic site. Paper points were transferred to the Anaerobe Reference Laboratory in the Department of Microbiology, Faculty of Medicine. They were stored frozen at −80 ºC within 5 min of sample collection until processed with PCR.

### DNA extraction

DNA was extracted from patients’ samples, cultured organisms and bacterial reference strains using QIAamp DNA Mini Kit (Qiagen, Hilden, Germany) according to the manufacturer’s protocols. For Gram-positive bacteria, the samples were incubated with a lysozyme solution before continuing with the rest of the protocol. The following positive controls were included in each PCR run: *P. melaninogenica* ATCC 25845, *F. nucleatum* ATCC 25586, *P. gingivalis* ATCC 33277*, T. forsythia* ATCC 43037, *T. denticola* ATCC 35405, *A. actinomycetemcomitans* ATCC 33384 and *P. micra* ATCC 33270.

### Conventional PCR and sequencing

Conventional PCR assays were used to detect the presence of *Fusobacterium* spp., *Prevotella* spp., *A. actinomycetemcomitans *, *P. micra *, *P. gingivalis *, *T. forsythia* and *T. denticola* using published primers shown in Table 2. Sequencing of the amplicon of *Prevotella* spp. was performed using a GenAmp PCR system 9700 by cycle sequencing with BigDye terminator (AB Applied BioSystems, Carlsbad, CA, USA).

**Table 2.** The primer’s sequences, product sizes, target genes and references for PCR assay

Test species	Primers′ sequences 5′−3′	Target genes	Product size	Reference
* A. actinomycetemcomitans *	F-: GGRAGAATGGATGGCGATAT R-: ATCAGAATGAACATAACCTATACCA	*hgpA*	81 bp	[4]
* P. micra *	F-: TGAGCAACCTACCTTACACAG R-: GCCCTTCTTACACCGATAAATC	16S rRNA	112 bp	[19]
* P. gingivalis *	F-: ACGAATCAAAGGTGGCTAAGTT R-: TTAGTCGCATTTTCGGCTGAT	*fimA*	85 bp	[19]
* T. forsythia *	F-: GATAGGCTTAACACATGCAAGTC R-: GTTGCGGGCAGGTTACATAC	16S rRNA	99 bp	[19]
* T. denticola *	F-: GGGCGGCTTGAAATAATRATG R-: CTCCCTTACCGTTCGACTTG	16S rRNA	92 bp	[19]
* Fusobacterium* spp.	F-: CGCAGAAGGTGAAAGTCCTGTAT R-: TGGTCCTCACTGATTCACACAGA	23S rRNA	101 bp	[20]
* Prevotella* spp*.*	F-: ACCAGCCAAGTAGCGTGCA R-: TGGACCTTCCGTATTACCGC	16S rRNA	153 bp	[21]

### Semi-quantitative PCR assay

A serial dilution of the amplicon of *P. melaninogenica* ATCC 25845 was done and the DNA concentration was calculated using nanodrop ND-8000. Agarose gel image was captured by MultiDoc-It Imaging System (UVP). By using a GS-800 calibrated densitometer (BioRad, Munich, Germany), the density of PCR band was measured for each dilution by using Quantity One software (BioRad). A standard curve was constructed by plotting the DNA concentration of the PCR band density. The DNA concentration for each sample was determined by extrapolation to the standard curve.

### Statistical analysis

For the results of PCR, the target anaerobes were tested using the *Z*-test to determine whether the patients with chronic periodontitis differ significantly from the healthy individuals. A level of *P*<0.05 was considered as statistically significant. For semi-quantitative PCR, values were presented as mean±sd, and unpaired *t*-test was used.

## Results

### Patients’ characteristics

The demographic characteristics of the patients and controls are shown in Table 3. There were more male patients than females with a ratio of 2 : 1 but the male-to-female ratio for the controls was 1 : 3.4. The patients’ ages ranged from 20 to 69 years (average=41.2 years). The majority of the MCP patients (10; 58.8 %) were in the age groups 20–39 years while 6 (46.2 %) with SCP were in the 50–59 age group. The distribution of the patients into these age strata did not attain any statistical significance, *P*>0.05. Among the CP patients, 23 (76.7 %) were non-Kuwaitis versus seven (23.3 %) Kuwaitis, *P* <0.05. Analysis of the patient’s nationalities showed that about half of the non-Kuwaiti patients (15 : 50 %) and controls (15 : 48.4 %) were Asians. Twenty-one (70 %) of the patients and 30 (96.8 %) of the controls were non-smokers. Five (62.5 %) of the eight smokers were patients with SCP compared with 2 (25 %) MCP patients and one (12.5 %) of the control group (*P <*0.002). Eleven (36.7 %) patients had comorbidities and versus none of controls, *P<*0.005.

**Table 3.** Demographic characteristics of the patients and controls

Characteristics	No. of individual patients and controls		
	Periodontitis patients (*n*=30)		Controls (*n*=31)
	Moderate (*P* value) (*n*=17)	Severe (*P* value) (*n*=13)	
Gender:			
Female	8 (<0.05)*	2 (<0.001*)	24
Male	9 (<0.05)*	11 (<0.001*)	7
Age (years)			
Mean+/-sd	39.6+/-3.2 (<0.0001)*	42+/-3.6 (<0.0001*)	32.5+/-1.7
Nationality:			
Kuwaiti	4 (>0.5)	3 (>0.5)	12
Non-Kuwaiti	13 (>0.5)	10 (>0.5)	19
Smoking Hx:			
Yes	2 (>0.5)	5 (<0.05)*	1
No	15 (>0.05)	6 (<0.0001)*	30
Ex-smoker	0 (<0.0001)*	2 (<0.05)*	0

*A significant *P* value.

### Detection of target anaerobes by PCR

The detection of the target anaerobes by PCR is shown in Table 4. Among the 17 MCP and 13 SCP patients, seven (41.7 %) and two (15.4 %), respectively, versus five (16.1 %) of 31 controls were positive for *A. actinomycetemcomitans* by PCR (*P>*0.05). The following anaerobes were also detected in patients and controls: *P. micra* in 16 (94.1 %) and 11 (84.6 %) patients with MCP and SCP, respectively, versus 16 (51.6 %) controls (*P*<0.0001 and *P*<0.0001); *P. gingivalis* 5 (29.4 %) and 5 (38.5 %), respectively, compared with two (6.4 %) controls, (*P*<0.0001 and *P*<0.0001); *T. forsythia* in 13 (76.5 %) and 12 (92.3 %), respectively, versus 16 (51.6 %) controls, (*P*<0.0001 and *P*<0.0001) and *T. denticola* in eight (64.7 %) and ten (76.9 %), respectively, versus nine (29 %) controls, (*P*<0.0001 and *P*<0.0001). *Fusobacterium* spp*.* were detected in all (100 %) patients and 29 (93.5 %) controls by PCR. Fifteen (88.2 %) MCP patients and 12 (92.3 %) SCP patients versus 30 (96.7 %) controls were positive for *Prevotella* spp. (*P*>0.05).

**Table 4.** Distribution of the target anaerobes detected by PCR from the patients and controls

Target anaerobes	No. (%) of patients/controls positive		
	Moderate (*n*=17)	Severe (*n*=13)	Control (*n*=31)
* Aggregatibacter actinomycetemcomitans *	7 (41.17)	2 (15.4)	5 (16.1)
* Fusobacterium* spp.	17 (100)	13 (100)	29 (93.6)
* Parvimonas micra *	16 (94.17)	11 (84.6)	16 (51.6)
* Porphyromonas gingivalis *	5 (29.4)	5 (38.5)	2 (6.5)
* Prevotella* spp.	15 (88.2)	12 (92.3)	30 (96.7)
* Tannerella forsythia *	13 (76.47)	12 (92.3)	16 (51.6)
* Treponema denticola *	8 (64.7)	10 (76.9)	9 (29.0)

### Identification of *Prevotella* to species level

A total of 57 *Prevotella* spp. from both patients and controls were identified. The results of the DNA sequencing are shown in Table 5. Four species were identified unequivocally by sequencing. They are *P. buccae* from 1 (1.6 %), *P. intermedia* 1 (1.6 %), *P. nanceiensis* 1 (1.6 %) and *P. nigrescens* 52 (85.3 %); the latter accounted for 52 (85.3 %). The *Prevotella intermedia* and *P. nanceiensis* detected were from a patient with MCP and a patient with SCP, respectively. Two (3.5 %) of the *Prevotella* spp. could not be identified to a species level.

**Table 5.** Speciation of the *Prevotella* spp. by sequence analysis

* Prevotella* spp.*	No. of periodontitis patients		No. of healthy controls	Total no. (*n*=61)
	Moderate (*P* value)†	Severe (*P* value)†		
* Prevotella buccae *	0	1 (>0.05)	0	1
* Prevotella intermedia *	1 (>0.05)	0	0	1
* Prevotella nanceiensis *	0	1 (>0.05)	0	1
* Prevotella nigrescens *	14 (>0.05)	8 (<0.05)†	30	52
* Prevotella* spp.	0	2 (<0.05)†	0	2
Total	15	12	30	57

*The GenBank references for identification are: LT707625.1, MT505539.1, GU561341.1, MT436583.1, and JF833152.1.

†*P* value is significant.

### Comparison of DNA concentration of *Prevotella* spp. in healthy individuals and patients

**Fig. 1.**
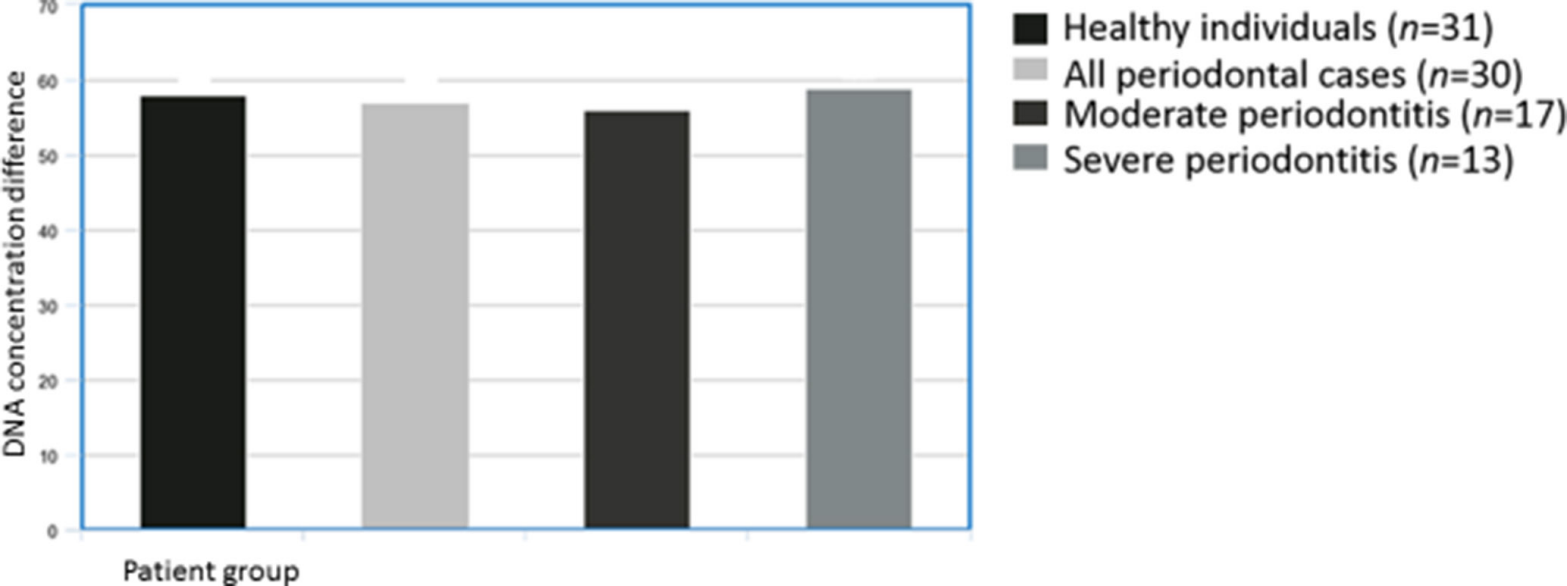
Comparison of DNA concentration of *Prevotella* spp. in healthy individuals, moderate periodontitis and severe periodontitis.

The differences between mean DNA concentrations of *Prevotella* spp. in healthy controls and CP patients were not statistically significant (*P* >0.5, Fig. 1).

## Discussion

In this study, the association between chronic periodontitis and seven putative anaerobic bacteria often associated with chronic periodontitis was studied in adult patients using molecular methods. In order to validate the results, an almost equal number of healthy individual volunteers was used as controls. The target patients were adults aged between 20–70 years because according to a meta-analysis report, patients with periodontal diseases are usually seen in adults with mean age of 44.16±8.35 rather than in younger patients [6]. It is assumed that elderly patients usually have other underlying diseases (e.g. diabetes mellitus) that can worsen periodontitis.

In this study, the prevalence of moderate and severe periodontitis was 56.7 and 43.3 %, respectively, a finding much higher than the 30.0 and 8.5 %, respectively, reported in the USA by the National Health and Nutrition Examination Survey (NHANES) [7]. Our data, though restricted to one single centre, also showed that Health Sciences Center, Kuwait has more patients with all forms of chronic periodontitis than American adults. However, we take cognizance of the fact that unless a larger population of patients with chronic periodontitis is studied this comparison may not be totally valid.

The majority of our patients as well as the controls were non-Kuwaitis. This is perhaps skewed by the disproportionately high expatriates living and working in Kuwait as over 3 million out of 4.2 million people in Kuwait are expatriates. The study also showed that there were more male patients than female by a ratio of 2 : 1. This finding is concordant with the data reported by Eke *et al*., in 2012 [7]. One of the important factors associated with CP is smoking. While the majority of the patients and controls did not smoke, a relatively large proportion (38.5 %) of the smokers, in this study, were patients with SCM, a highly significant finding (*P*=0.00188) similar to that previously reported by other groups in Atlanta [7].

In our study, *P. gingivalis* was detected in a relatively low proportion (33.3 %) of patients with CP. This is much lower than the high figure of 83.6 % reported by Faghri *et al.* [8]. The reason for this disparity is unclear at this time but it may be partly attributable to variations in sampling techniques and DNA extraction. For example, leaving the paper-points in the pockets for 10 s might have been too short (better if it would be 29–30 s). The second explanation is that during sampling excessively blood-soaked points, samples were discarded and new samples taken from a less haemorrhagic site and this could also be a cause for lower *P. gingivalis* proportions, since *P. gingivalis* proliferates and prefers blood environments. It is conceivable, although debatable, that *P. gingivalis* is the most important bacterium causing CP in adult patients attending the DC of KUHSC. This assertion is supported by its strong statistically significant association with moderate and severe CP. A similar finding to ours has been published before in a meta-analysis reported by Refiei *et al.* [6]. In their report, the prevalence of *P. gingivalis* in patients with CP was significantly higher than in healthy subjects. However, in contrast to the reports published by Al-hebshi *et al.* [4], and Kumar *et al.,* [9], which categorically singled out *P. gingivalis* as the keystone bacteria causing CP, our data in this study did not seem to unequivocally back that claim.

In our study*, A. actinomycetemcomitans* was detected in a significantly high proportion of MCP cases but in a low proportion of the SCP cases. Our figures ‘41.7 and 15.4 %’ are much higher than those reported in the study of Cortelli *et al.* [10]. They demonstrated the occurrence rates of 17.4 and 23.1 % in their patients with MCP and SCP, respectively. However, this bacterium has been reported in other studies at much higher detection rates of 92.4 % [11]. Very high statistically significant proportions of the patients with MCP (96.4 %) and SCP (84.6 %) compared with the controls (*P* <0.001) were positive for *P. micra *, a finding similar to that reported in a sister GCC country, Yemen [4]. However, some studies have also reported much lower figures (58–63 %) than ours but by culture technique [12]. The proportion of our patients from whom *T. forsythia* was detected was very high across both spectra of the disease. In support of our findings, as reported previously by Deng *et al.* [13], *T. forsythia* has been positively associated with CP in 77.1 % of CP patients. However, in contrast, there are other studies in the literature [12] that have reported a much lower detection rate than ours and Deng’s [13].

Contribution of *Prevotella* spp. to the etiological agents of CP, in this study, appeared to be negligible as PCR positivity was almost the same and in very high proportion of the patients and controls and therefore could be deemed to be the most common member of the anaerobic normal microbiota of the oral cavity. These results are consistent with and supported by the conclusions of previous studies by Aas *et al.* [14], and Dewhirst *et al.* [15], who affirmed that *Prevotella* spp. are part of the normal flora in the oral cavity. But in an earlier study by Dorn *et al.,* [16] it was reported that *P. intermedia* was capable of invading the oral epithelial cells and causing periodontitis. The most common species, in our study, was *P. nigrescens,* which was detected in the majority of patients and healthy control individuals using DNA sequencing. It was established that patients with CP and healthy control individuals harboured the same variety of *Prevotella* spp. A previous report by Rotimi *et al.* [17] also showed that *P. nigrescens* was the most prominent anaerobic microbiota in the saliva of healthy children in Kuwait. In an attempt to demonstrate if there is a variation in the quantity of *Prevotella* spp. at infected and normal sites, and demonstrate this as a potential pathogenic factor, a semi-quantitative PCR assay was used to determine the concentration of DNA extracted from the species at these sites. In our hands, there was no significant difference in their concentrations at these sites. This lead us to speculate, at this time, that *Prevotella* spp. may not contribute to the pathogenesis of periodontitis and may not be regarded as one of the putative anaerobic target bacteria that cause CP in Kuwait, although the majority of the population in Kuwait are non-Kuwaiti.

Previous studies [3, 18], observed five and six major complexes in different subgingival and supragingival plaque samples using whole-genomic DNA probes and checkerboard DNA–DNA hybridization, respectively. In our study, subgingival plaque samples were studied using PCR that targeted seven specific anaerobes. In our study, we could detect complex 1 (i.e. *P. gingivalis *, *T. forsythia* and *T. denticola *), two complex (* Fusobacterium* spp.*, Prevotella* spp.*, P. micra*) and five (* A *. *actinomycetemcomitans *) organisms.

This study is not without its limitations. The small number of patients and the use to a single centre to conduct the study might lead to underrepresenting the real situation in Kuwait. A larger sample size would have been better and would have provided a more accurate picture of the anaerobic etiological agents of CP in Kuwait. However, CP does not appear to be a big problem in Kuwait as only 30 patients met the inclusion criteria over a period of 15 months. In spite of this draw back, it is important to point out that some studies have even used a smaller number of patients than ours with almost the same conclusions. For example, in the study by Al-hebshi *et al.,* [4] only 20 patients with periodontitis were recruited and periodontitis sites were compared with healthy sites in the same patients. The semi-quantitative PCR method has its limitation as well. For example, the procedure depends on the PCR gel image and sometimes the weak bands may not be detected by the software. Because it is a very sensitive method, even changing the paper used for capturing the PCR gel image may affect the results.

## Conclusion

It is noteworthy that *P. gingivalis* was found in MCP and SCP periodontitis in moderate proportions and in small proportion of the controls while *T. forsythia, P. micra* and *T. denticola* were found in high proportions in moderate and severe cases compared to controls; these differences were statistically significant. In this study, *A. actinomycetemcomitans* was detected in higher numbers in the moderate cases than the controls, a finding that was statistically non-significant. *Prevotella* DNA sequencing results showed that 91.2 % of the *Prevotella* spp. were *P. nigrescens* and were detected in both patients and controls alike, throwing into doubt its pathogenic potential in chronic periodontitis.
